# The differential role of IL-33 and IL-38 in prostate cancer, contradictory roles

**DOI:** 10.3389/fimmu.2025.1623038

**Published:** 2025-06-18

**Authors:** Runfen Ban, Shanshan Gao, Bin Zhou, Shisan Bao

**Affiliations:** ^1^ Department of Urology, Gansu Provincial People’s Hospital, Lanzhou, China; ^2^ Haemodialysis Centre, Gansu Provincial People’s Hospital, Lanzhou, Gansu, China; ^3^ Scientific Research Division, The First People’s Hospital of Baiyin, Gansu, China

**Keywords:** IL-33, IL-38, prostate cancer, precision medicine, diagnosis

## Abstract

Prostate cancer (PCa) is still as a major cause of morbidity and mortality in men at the global level, highlighting the necessity for improved diagnostic and therapeutic strategies beyond current PSA screening limitations. This mini-review focuses on the complex and often opposing roles of two key cytokines, IL-33 and IL-38, within the tumour microenvironment and their implications for host immunosurveillance in PCa. Intra-tumoral IL-33 expression is significantly reduced in PCa tissues and correlates with aggressive disease features such as higher Gleason scores and lymphatic metastasis, suggesting an inherent anti-tumour function. Such a protective role may be mediated *via* the ST2/NF-κB signalling pathway and the recruitment of lymphocytes into the tumour microenvironment. However, a paradoxical increase in circulating IL-33 levels in PCa patients hints at complex systemic compensatory mechanisms or differential compartmental regulation. In contrast, intra-tumoral IL-38 exhibits markedly elevated expression in PCa compared to benign prostatic hyperplasia and non-cancerous tissues. This increased IL-38 correlates with tumour severity, advanced TNM stages, and poorer overall survival, indicating a pro-tumoral role. Mechanistically, IL-38 appears to inhibit CD8^+^ cytotoxic T cell infiltration and potentially promotes immunosuppression through the upregulation of regulatory T cells (Tregs), thereby facilitating tumour progression. The contrasting expression patterns and clinicopathological associations of IL-33 and IL-38 highlight their potential as novel biomarkers for PCa diagnosis and prognosis. Further comprehensive investigation, including multi-centre studies across diverse populations, functional *in vitro* and *in vivo* analyses, and exploration of their therapeutic targetability, is crucial to translate these findings into effective precision medicine strategies for PCa patients.

## Introduction

Despite extensive basic and clinical research and significant advancements in medical interventions for prostate cancer (PCa) (1), PCa remains the second most common cancer and the fifth leading cause of cancer-related deaths among men globally (2). The global burden is considerable, with approximately 1.4 million cases and 375,000 deaths reported annually (3). This persistent morbidity and mortality are attributed to an ageing population, environmental pollution from industrialisation, and lifestyle shifts such as reduced physical activity (3).

Late diagnosis significantly compromises the overall five-year survival rate, particularly dropping below 30% in cases with distant metastases. In contrast, patients with localised PCa (approximately 70% of cases) generally exhibit more favourable outcomes following appropriate treatment (1).

Currently, prostate-specific antigen (PSA) screening remains the mainstay for early detection. However, while sensitive, PSA lacks specificity, limiting its effectiveness for early diagnosis and precise clinical decision-making (4). Therefore, the identification of novel biomarkers with both high sensitivity and specificity is urgently needed to enable early detection and personalised treatment strategies.

## Host immunity and prostate cancer

Host immunity plays a fundamental role in cancer immunosurveillance. Both innate and adaptive immune responses are essential for modulating tumour initiation, progression, and metastasis (5). Chronic local inflammation and persistent immune suppression are well-established drivers of PCa development (6). Disruption of immune surveillance enables transformed cells to evade detection and progress into malignant tumours (7), involving key immune effectors such as CD8^+^ cytotoxic T cells, CD4^+^ helper T cells, and CD25^+^ regulatory T cells (Tregs).

Interactions between tumour cells and the surrounding microenvironment critically shape PCa progression. Pro- or anti-inflammatory cytokines modulate these interactions, influencing host immunity and tumour outcomes (6). Thus, a deeper understanding of these immunological kinetics is crucial for improving immunotherapeutic strategies (8). Identifying cytokines with prognostic and/or therapeutic significance could improve clinical outcomes via reshaping the tumour microenvironment.

## IL-33 and prostate cancer

IL-33, a member of the IL-1 cytokine superfamily, was first identified in 2003 by Baekkevold et al. (9). The biologically active form (~30 kDa) is predominantly associated with Type 2 immunity and is commonly expressed in keratinocytes, endothelial cells, and fibroblasts (10). Its biological effects are mediated through its receptor ST2, expressed on various leukocytes including group 2 innate lymphoid cells (ILC2s), Tregs, and mast cells (11).

Interestingly, IL-33 can promote either Type 1 or Type 2 immune responses, depending on the microenvironment (11). It induces NF-κB activity and pro-inflammatory cytokine release *via* the ST2 pathway (12), and its expression is typically upregulated in chronic inflammatory conditions such as inflammatory bowel disease (IBD) and rheumatoid arthritis (13). IL-33 is released in response to mechanical injury or necrosis, acting as an alarmin.

Wang et al. explore IL-33 expression in PCa using bioinformatics analyses (TCGA, TIMER, HPA databases), followed by validation in clinical samples (PCa, benign prostatic hyperplasia [BPH], and non-cancerous tissues) (14). Immunohistochemistry confirms significantly reduced IL-33 expression in PCa tissues. This reduction of intra-tumoral IL-33 may impair anti-tumour immune surveillance by weakening the Th1 response, thereby promoting tumour development.

In addition to studies conducted in Chinese populations (14), research from a Canadian cohort has demonstrated that IL-33 expression is significantly reduced in metastatic prostate cancer (PCa) compared to primary PCa or benign prostatic hyperplasia (BPH) (15). A similar pattern is observed for the HLA-A, HLA-B, and HLA-C genes, which are downregulated in metastatic castration-resistant PCa but upregulated in high-risk, neo-hormone-treated primary tumours. These findings suggest that IL-33 may play a role in maintaining an immune-recognisable phenotype in primary tumours. In human PCa, IL-33 and MHC-I/HLA genes are co-ordinately downregulated during metastatic reprogramming, indicating a potential role for IL-33 in tumour progression and immune evasion (15). Further validation of IL-33’s role in immune escape and tumour progression is needed using both human PCa samples and relevant animal models.

Further analysis reveals decreased expression levels of ST2 and NF-κB in PCa, suggesting that IL-33 downregulation occurs *via* the ST2/NF-κB axis. Notably, lower IL-33 expression correlated with higher Gleason scores and lymphatic metastasis, highlighting its anti-tumour role. However, these observations are based on eye visual scoring methods prone to bias. Automated image analysis would improve objectivity and reproducibility in future study (16–19).

IL-33 levels are positively correlated with lymphocyte infiltration, suggesting a protective role in immune recruitment. Interestingly, IL-33 expression is lower in Caucasian patients compared to non-Caucasians, while no significant age-related differences are detected. Region-specific differences in IL-33 expression within the prostate are also noted—levels are lower in the peripheral zone, consistent with poorer prognosis in cancers arising from this region (20). IL-33 expression also positively correlated with CD68^+^ macrophage infiltration.

In summary, IL-33/ST2/NF-κB expression is reduced in PCa and associated with immune cell infiltration, tumour grade, TP53 mutations, and metastasis. These findings support an anti-tumour role for IL-33 *via* the ST2/NF-κB pathway.

Paradoxically, Chatrabnous et al. report increased circulating IL-33 levels in PCa patients, correlating with higher Gleason scores (21). This discrepancy—reduced intra-tumoral versus elevated systemic IL-33—may reflect differential compartmental regulation. Intra-tumoral IL-33 suppression may compromise local immune surveillance, while systemic IL-33 could represent a compensatory response to tumour progression, potentially influenced by host genetics such as IL-33 SNPs (e.g., rs1929992) (21–23).

This hypothesis warrants further investigation using multi-centre studies across diverse populations and paired analyses of intra-tumoral and circulating IL-33 levels. Animal models with genetic manipulation of IL-33 and ST2, as well as *in vitro* studies, could elucidate mechanisms relevant to precision medicine.

## IL-38 in prostate cancer

Intra-tumoral expression of IL-38 is markedly elevated in PCa tissues compared to non-cancerous or benign prostatic hyperplasia (BPH) tissues (18). IL-38 is primarily localised in the nuclei, with a smaller proportion present in the cytoplasm. The precise underlying mechanism remains unclear and requires verification through molecular biology and/or bioinformatics approaches. Importantly, the specificity and sensitivity of intra-tumoral IL-38 have been validated using ROC curve analysis, suggesting that IL-38 is a reliable biomarker. Additionally, intra-tumoral IL-38 expression correlates with both the Gleason score (24) and circulating PSA levels (18), suggesting its association with tumour severity and disease progression.

This concept is further supported by the observation that higher IL-38 levels are associated with advanced TNM stages, particularly those involving distant metastasis. Moreover, IL-38 expression has been linked to prognostic value (18). As an anti-inflammatory cytokine, IL-38 plays a key role in maintaining physiological homeostasis and suppressing inflammation, particularly in autoimmune diseases. Its contribution to PCa progression aligns with findings in non-small cell lung cancer (NSCLC), where an inverse correlation has been observed between IL-38 expression and tumour differentiation (25), suggesting a pro-tumoral role for IL-38. This is further supported by *in vivo* studies using IL-38-plasmid-transfected Lewis lung carcinoma cells, which developed significantly larger tumours compared to controls (26). Notably, the increased tumour size was inversely correlated with infiltrating CD8^+^ cytotoxic T cells, suggesting that IL-38 may suppress host cellular immunity *via* upregulating Treg cells (27). By contrast, intra-tumoral IL-38 is inversely correlated with differentiation and invasion in colorectal cancer (19), suggesting IL-38 plays a protective role during malignancy development. The discrepancy observed between PCa and colorectal cancer may be due to the different microbial load**;** prostate tissue is almost germ-free, whereas the colon contains almost 10^14^ microbiota. These distinct micro-environments could trigger substantially different host responses during the development of malignant tumours, resulting in differential local IL-38 expression.

To investigate the potential immunological mechanisms, the relationship between IL-38 expression and lymphocyte subsets has been determined. An inverse correlation is observed between IL-38 and infiltrating CD8^+^ T cells in PCa tissues (18), suggesting that IL-38 may promote tumour progression by inhibiting CD8^+^ T cell infiltration, either directly or indirectly. The relationship between IL-38 production and CD8^+^ T cell mediated cytotoxicity in PCa remains to be explored. While PD-1 is well known to contribute to tumour immune evasion (28), an unexpected inverse correlation between intra-tumoral IL-38 and PD-1 has been detected. This is paradoxical, given that IL-38 appears to be pro-tumoral, whereas PD-1 is typically associated with immunosuppressive tumour environments (18).

Interestingly, no significant correlation is found between IL-38 and infiltrating CD4^+^ helper T cells or CD20^+^ B cells in PCa tissues, indicating that IL-38 may differentially regulate various lymphocyte subsets and exert distinct effects on cellular versus humoral immune responses. These differences may reflect variations in the tumour microenvironment and warrant further investigation.

Identifying the cellular source of intra-tumoral IL-38 is critical for therapeutic development. Studies indicate that IL-38 in PCa originates from tumour epithelial cells, CD138^+^ plasmacytes, CD3^+^ T cells, and CD68^+^ macrophages (18). While IL-38 is traditionally considered to derive from leukocytes and keratinocytes (29), findings by Wu et al. demonstrate tumour epithelial cells as a major source in PCa (18). Such findings further suggest a pro-tumoral role for IL-38 during PCa development, and that IL-38 functions in both a paracrine and autocrine manner in PCa.

The exact mechanism by which tumour epithelial cells secrete IL-38—whether through paracrine or autocrine signalling—remains to be elucidated. However, it is hypothesised that elevated IL-38 may initially suppress inflammation *via* Tregs, as part of a response to chronic inflammation in the prostate (30). This immune suppression may weaken host defences, facilitating immune escape of transformed cells, particularly under persistent stressors such as smoking (31).

As the disease progresses, transformed prostate epithelial cells appear to produce IL-38, enhancing local immunosuppression. This supports a transition from paracrine to autocrine action. Consistent with this, highly proliferative PCa cells have been shown to produce nearly twice as much IL-38 as less proliferative cells (18). Anti-IL-38 therapy has been reported to restore host anti-tumour immunity by counteracting tumour-induced suppression of early immune activation in mouse models of breast cancer and melanoma (32), emphasising its potential in precision oncology.

Importantly, IL-38 expression correlates with survival outcomes. Patients with low intra-tumoral IL-38 exhibit significantly longer overall survival, including those with advanced disease (prognostic stages III–IV or Gleason score ≥8) (18). Interestingly, this association is observed in stages I–III but not in stage IV, where IL-38 levels decline. This decrease may result from extensive tissue damage or compression in the prostate, impairing IL-38 production, though this hypothesis requires further study.

Cox regression analysis identified IL-38 expression, PSA level, T stage, TNM stage, and prognostic stage as significant predictors of patient survival in univariate analysis. Multivariate analysis further confirms IL-38 and TNM stage as independent prognostic indicators (18), suggesting that intra-tumoral IL-38 may serve as a useful biomarker for both diagnosis and targeted therapy.

However, several factors—including age, serum PSA, recurrence risk, prognostic grouping, lymph node metastasis, CD8 and PD-1 levels, and combined IL-38 with CD8 or PD-1—are not significantly associated with survival outcomes (18). PSA’s limitations as a specific diagnostic tool are well known (4). The lack of age correlation likely reflects the advanced age of most PCa patients, who may no longer experience androgen-driven tumour growth. Surprisingly, IL-38 showed no significant association with recurrence or prognosis of PCa. Similarly, the lack of correlation with lymph node metastasis suggests either a minimal role for IL-38 in PCa cell migration or an effect too subtle to detect. Interestingly, IL-38 has been linked to metastasis in colorectal cancer animal models (33), possibly reflecting species-specific or tumour-type differences.

The absence of significant correlation between IL-38 and the combined CD8^+^/PD-1 axis implies that IL-38 may not act primarily through the PD-1/PD-L1 pathway in PCa. Alternatively, limited sample size may have introduced bias, highlighting the need for larger, multicentre studies. Moreover, the lack of correlation with CD8^+^ T cells may suggest that IL-38, as an anti-inflammatory cytokine, that more potently influences CD4^+^ T cells, particularly Tregs (27). This should be explored in future, using genetically modified IL-38 animal models (34).

There are distinct microenvironmental differences between colon and prostate tissues—most notably in their resident microbial communities. The colon harbours a dense and diverse microbiota, whereas the prostate contains relatively few microbial inhabitants. Even a small number of unusual microorganisms in the prostate can lead to chronic prostatitis, creating a persistent inflammatory environment that may contribute to the development of PCa. These disparities may significantly influence local immune responses and each tissue’s capacity to maintain homeostasis, potentially affecting carcinogenesis. Further studies are warranted to elucidate these mechanisms, including investigations using animal models and analyses of human tissue samples. Future research should also aim to characterise the functional impact of specific microbial taxa on immune modulation and tumour development in these distinct tissue environments.

Given that intra-tumoral IL-38 serves as a robust prognostic indicator, it holds promise for incorporation into clinical diagnostic and therapeutic protocols.

As stated above, PSA is well recognised as a useful diagnostic tool in clinical practice (4). However, a direct comparison of circulating levels of PSA, IL-33, and IL-38 would be highly valuable for assessing the potential clinical utility of IL-33 and/or IL-38 in early diagnosis or as therapeutic targets. This hypothesis should be tested in future studies.

The IL-1 cytokine family comprises a diverse group of both pro-inflammatory and anti-inflammatory mediators, including IL-1α, IL-1β, IL-18, IL-33, IL-36α/β/γ, IL-37, and IL-38. In this mini-review, we compared the roles of IL-1β and IL-36 with those of IL-33 and IL-38 in prostate cancer (PCa). Although these cytokines belong to the same family, they exhibit distinct roles in carcinogenesis.

An inverse association between androgen receptor (AR) expression and IL-1β has been observed in a cohort of patients with metastatic castration-resistant prostate cancer (35). IL-1β plays a key role in promoting skeletal metastasis by shaping the metastatic microenvironment to support disease progression. Notably, the current standard of care for PCa, which involves inhibition of the AR signalling axis in tumour cells, may inadvertently lead to increased IL-1β production (35).

Despite an extensive literature search, there is limited evidence on the role of IL-36 in prostate cancer. However, based on findings in other malignancies, such as colorectal cancer (36), and the fact that IL-38 binds to the IL-36 receptor and exhibits immune-regulatory effects similar to the IL-36 receptor antagonist (37), we speculate that IL-36 may promote PCa progression through its pro-inflammatory activity—potentially by enhancing M1 macrophage polarisation. This hypothesis warrants further investigation in future studies.

## Conclusion

Differential expression of intra-tumoral IL-33 and IL-38 emphasises the importance of host immunosurveillance in PCa development. Targeting IL-33 and/or IL-38 may enhance precision medicine strategies and improve clinical outcomes for PCa patients. However, their diagnostic and prognostic utility still requires further validation.
